# Preparation of Tung Oil Microcapsules Coated with Chitosan–Arabic Gum and Its Effect on the Properties of UV Coating

**DOI:** 10.3390/polym17141985

**Published:** 2025-07-19

**Authors:** Jinzhe Deng, Xiaoxing Yan

**Affiliations:** 1 Co-Innovation Center of Efficient Processing and Utilization of Forest Resources, Nanjing Forestry University, Nanjing 210037, China; dengjinzhe@njfu.edu.cn; 2 College of Furnishings and Industrial Design, Nanjing Forestry University, Nanjing 210037, China

**Keywords:** microcapsules, UV coatings, self-repairing

## Abstract

Tung oil, as dry oil, can quickly dry and polymerize into tough and glossy waterproof coatings, with a very high application value. Tung oil was used as a core material to prepare Tung oil microcapsules coated with chitosan–Arabic gum, and the preparation process of the microcapsules was optimized. The effect of adding a UV coating on the performance of the microcapsules was explored. Under the conditions of a core–wall mass ratio of 0.5:1.0, pH value of 3.5, mass ratio of chitosan to Arabic gum of 1.0:4.0, and spray drying temperature of 130 °C, Tung oil microcapsules coated with chitosan–Arabic gum had a higher yield and coverage rate, which were 32.85% and 33.20%, respectively. With the increase of the spray drying temperature during preparation, the roughness of the coating first increased and then decreased, the visible light transmittance decreased first and then increased, and the glossiness showed an overall downward trend. The self-repairing rate decreased gradually. When the microcapsules #11 were added to the UV topcoat at 5%, the coating can obtain excellent comprehensive properties; the roughness was 0.79 μm, elongation at break was 5.04%, visible light transmittance was 77.96%, gloss loss rate was 10.95%, and self-repairing rate was 20.47%.

## 1. Introduction

With the continuous development of the furniture industry, people’s exploration of furniture materials has never stopped [1,2,3,4,5,6,7]. Wood is one of the most commonly used materials in making furniture [8,9,10]. In order to enhance the application effect of wood in furniture, the physical properties [11,12,13,14,15], manufacturing processes [16,17,18,19], coating properties [20,21,22,23,24], etc., of wood are all the focus of research in the field of furniture. After applying paint on the surface of furniture, the resulting coating can provide protection for the furniture. Due to the inevitable damage to the surface coating caused by daily friction, it is necessary to explore new ways to optimize the surface coating. As a new type of smart material, self-healing coatings have become one of the research hotspots [25]. By using microcapsule technology to improve the performance of the coating, microcapsules with self-repairing performance are added to the coating film. When the coating film is damaged, the core material of the microcapsules flows out to repair the wear of the coating in time [26,27]. Zhang et al. [28] successfully synthesized microcapsules containing 4, 4′-dicyclohexylmethane diisocyanate (HMDI) by using the integrated microcapsule method of electro-spray and interfacial polymerization. The results showed that HMDI could leak from the microcapsules and solidify, alleviating the corrosion hazard to the metal steel. Karampoor et al. [29] studied self-repairing coatings based on polyurea–formaldehyde microcapsules with flaxseed oil as the repairing agent. The multifunctional coating obtained in this way had a good self-repairing effect. Xia et al. [30] prepared microcapsules with melamine resin as a wall material and a UV topcoat as the core material and added them to the UV topcoat. The results showed that the addition of UV topcoat microcapsules could enhance the toughness of the UV topcoat film to a certain extent, inhibit the formation of micro-cracks, and have a good self-repairing effect. Currently, the research on self-repairing microcapsules mainly focuses on using resins synthesized by the condensation of melamine, formaldehyde, urea, etc., as the wall material. This material releases formaldehyde into the surrounding environment during preparation. With the increasing emphasis on green and environmental protection concepts in society, a material that does not produce harmful substances during preparation and use is needed as an alternative. Therefore, self-healing microcapsules prepared with natural materials as the wall and core materials have broad research and application prospects.

Chitosan is a natural cationic polysaccharide, commonly found in the exoskeletons of marine crustaceans and the cell walls of fungi. Due to its excellent biocompatibility and film-forming properties, it is widely used in various fields [31,32,33]. However, chitosan has poor interfacial polymerization characteristics, and its coverage rate for core materials is relatively low when used alone [34,35]. Therefore, natural anionic polysaccharide Arabic gum is combined with chitosan as the wall material for microcapsules. Butstraen [36] et al. successfully studied the encapsulation of a commercially available blend of triglycerides using a wall material of chitosan–Arabic gum. Baiocco [37] et al. also successfully prepared chitosan–Arabic gum-coated peppermint essential oil microcapsules. The European Patent Office also has relevant patents on Amal Elabbadi [38] et al.’s Arabic gum chitosan system. Since drying oils easily oxidize and dry in the air to form elastic and flexible solid films, they can be used as core materials for microcapsules without the need for a catalyst. When the microcapsules are damaged, the drying oil encapsulated within them is released, oxidizes, and dries to form a solid film, thereby repairing the damaged coating area [39]. Tung oil, as a drying oil [40], mainly consists of Tung oil acid glycerides. This highly unsaturated conjugated system is the main reason for the rapid polymerization and excellent drying performance of this oil [41]. Chang [42] et al. prepared self-healing microcapsules using chitosan as the wall material and wood wax oil as the core material, and the results showed that the microcapsules had effective repairing properties for scratches. The above indicates that chitosan–Arabic gum has a certain feasibility in being used as a wall material to prepare microcapsules with Tung oil as the core material. The self-repairing microcapsules were prepared using chitosan–Arabic gum as the wall material and Tung oil as the core material. The microcapsules were added to UV topcoats, and the comprehensive performance of the coatings was tested to explore the influence of the Tung oil microcapsules coated with chitosan–Arabic gum on the coating performance. This provides a certain technical basis for the preparation of UV coatings with self-repairing performance.

## 2. Materials and Methods

### 2.1. Materials

The various materials used in the preparation of the microcapsule samples are shown in Table 1. The coating used was a UV topcoat, provided by Jiangsu Haitian Technology Co., Ltd. (Taizhou, China). A 50 mm × 25 mm × 25 mm silicone mold was used when preparing the coating film. The glass panel specification is 75 mm × 25 mm. The equipment used in the experiment is shown in Table 2.

### 2.2. Preparation of Tung Oil Microcapsules Coated with Chitosan–Arabic Gum

The microcapsules were prepared using Arabic gum and chitosan as the wall materials, and Tung oil as the core material. A tannic acid was used for the solidification of the microcapsules. Because the Arabic gum has a good emulsifying effect, the core material lotion was prepared by dropping Tung oil directly into the Arabic gum solution. An acetic acid was added dropwise to change the pH value of the solution; the chitosan solution was positively charged and the Arabic gum solution was negatively charged. The two substances condensed into microcapsules due to electrical neutralization. Finally, the tannic acid was added to solidify the microcapsules.

Factor A, the core–wall mass ratio, factor B, the pH value during the complex coagulation reaction, factor C, the mass ratio of chitosan to Arabic gum, and factor D, the temperature during spray drying, were selected, and three levels of each factor were selected to design four-factor three-level orthogonal experiments. The specific experimental plan and details are shown in Table 3 and Table 4. The # in the table is used to number the microcapsule samples, where #1 represents the prepared microcapsule sample number 1.

The steps for preparing the microcapsules were as follows. Microcapsules #1 are used as an example.

According to the detailed list of experimental materials, 3.200 g of Arabic gum powder was weighed and it was added with deionized water into a beaker. The beaker was placed in a heat-collecting magnetic stirrer at 50 °C and 1000 rpm for 1 h, until all the Arabic gum powder was dissolved, to obtain a 4% Arabic gum solution. Then, according to a core to wall ratio of 0.5:1.0, 2.000 g of Tung oil was weighed and added to the beaker containing the Arabic gum solution. The heat-collecting magnetic stirrer was set to 2200 rpm and stirred for 1 h. After stirring, the beaker was placed into an ultrasonic emulsifier disperser and sonicated for 10 min under the condition of a 1 s intermittent ultrasound. The product was the core material emulsion.

A total of 78.404 g of water was weighed and placed in the beaker. Then, 0.796 g of acetic acid (concentration of 99.5%) was weighed and added to the beaker containing deionized water, stirring evenly. Then, 79.200 g of 1% acetic acid solution was prepared. A total of 0.800 g of chitosan was weighed and added to 79.200 g of 1% acetic acid solution. The beaker was placed in the heat-collecting magnetic stirrer at 50 °C and 1000 rpm to prepare a 1% chitosan solution. The 1% chitosan solution was dropped into the beaker of the core material emulsion. The pH of the solution was adjusted to 3 with the acetic acid. Then, it was placed in the heat-collecting magnetic stirrer set to heat and stir for 30 min at 50 °C and 800 rpm. Then, 160.000 g of deionized water was weighed and added to the beaker to dilute the solution. Subsequently, the beaker was placed in the heat-collecting magnetic stirrer at 25 °C and 800 rpm for a reaction of 30 min. To solidify the microcapsules, tannic acid was added to the solution and stirred continuously for 2 h. The obtained product was a microcapsule solution. The microcapsule solution was left at room temperature for 24 h before drying. The parameters of the spray dryer were set as a feeding speed of 200 mL/h and an inlet temperature of 150 °C. After the sample was dried, the powdered microcapsule samples were collected from the collector. The specific material details during sample preparation are shown in Table 5.

### 2.3. Preparation of UV Topcoat Film-Containing Microcapsules

The microcapsule sample prepared in the single-factor experiment was added to the UV topcoat at a content of 5% to prepare a UV topcoat film containing microcapsules. When preparing the UV topcoat film containing microcapsules, the UV topcoat and microcapsules were weighed separately in proportion, and the microcapsules and UV topcoat were added together to the beaker. The beaker was placed into the magnetic stirrer, and the stirring speed of the magnetic stirrer was set to 800 rpm with a stirring time of 15 min. Subsequently, the UV topcoat containing 5% microcapsules was obtained. The mixed coating of 1.0 g was weighed, and the mixed coating was applied to a glass plate. An 80 μm UV topcoat film was prepared by using a coating preparation device. The substrate was securely fixed on a leveled platform prior to using the coating preparation device. The coating preparation device was carefully aligned parallel to the substrate edge and slid over the surface at a constant speed to ensure coating uniformity. The UV topcoat film containing microcapsules was coated on the glass plate, levelled for 30 min, and then cured in the UV curing machine for about 60 s.

### 2.4. Test and Characterization

#### 2.4.1. Microcapsule Sample Characterization

Yield test: After the sample was dried, the mass of the microcapsules was recorded as *M_a_.* The quality of the raw materials used in the microcapsules’ preparation was recorded as *M_b_*. The ratio of the quality of the collected microcapsules to the quality of the feed was the yield of the microcapsules. The calculation formula of yield *Y* is shown in Formula (1).

*Y* = (*M_a_*/*M_b_*) × 100% (1)

Coverage rate test: The microcapsules of mass *M*_0_ were poured into a mortar and fully ground until the microcapsules’ wall material broke. The ground microcapsules were poured into a beaker, and a certain amount of ethanol was added to the beaker so that the microcapsules were fully soaked. After 48 h, the microcapsules were filtered by a vacuum filter. During the filtration process, the microcapsules were continuously washed to separate the residual core material from the wall material. After extraction and filtration, the product was put into a blast drying oven at 50 °C to dry to constant weight, and the weight was recorded as *M*_1_. The coverage rate *C* was calculated as shown in Formula (2).

*C* = [(*M*_0_ − *M*_1_)/*M*_0_] × 100% (2)

Morphology characterization: An SEM QUANTA 200 was used to analyze the morphology and particle size of the microcapsules.

Chemical composition characterization: A Fourier infrared spectrometer VERTEX 80V was used to analyze the chemical composition in the microcapsules and UV topcoat films.

#### 2.4.2. UV Topcoat Performance Tests

Self-repairing performance test of UV topcoat film: A scratch test was used to test the self-repairing performance of the UV topcoat film. A single-sided guard blade (38 mm × 19 mm) was used for uniform speed across the coating. A scratch of about 15 mm was left on the coating. An optical microscope was used to observe and record the width of the scratches. The width of the scratches was recorded as *W*_1_. Then, the scratch was observed at that location again after 48 h and the scratch width was recorded as *W*_2_. The self-repairing rate of the UV topcoat film was calculated from *W*_2_ and *W*_1_. The calculation formula for the self-repairing rate *R* is shown in Formula (3).

*R* = [(*W*_1_
*− W*_2_)/*W*_1_] × 100% (3)

Roughness test of UV topcoat film: When testing the roughness of the UV topcoat film, the fine roughness tester J8-4C was used. The sample was placed on a sample stage and the testing and recording of data started when the probe came into contact with the surface of the sample.

Breaking elongation test of UV topcoat film: The UV topcoat mixed with microcapsules was poured into a silicone mold, cured, and demolded. During testing, both ends of the UV topcoat film were clamped in the fixture of the universal mechanical testing machine. The speed during stretching was set to 0.5 mm/min, and the sample was stretched to fracture. The calculation method for the breaking elongation of the UV topcoat film is shown in Formula (4), where *e* represents the breaking elongation of the UV topcoat film at the point of rupture, *L*_0_ is the initial distance between the upper and lower clamping arms when the UV topcoat film is tightened, and *L*_1_ is the distance between the upper and lower clamping arms when the UV topcoat film breaks.

*e* = [(*L*_1_ − *L*_0_)/*L*_0_] × 100% (4)

Transmittance test of UV topcoat film: Transmittance is the ability of light to pass through a sample, and its optical properties are characterized by measuring the transmittance of the sample to a specific wavelength of light. The light transmittance of the prepared film was the percentage of the light flux passing through the sample and the incident light flux. The UV spectrophotometer U3900 was used to test the light transmittance of the coating with and without microcapsules. Specular geometry was adopted for the optical path setup in the transmittance measurements. During sample preparation, the UV topcoat film was evenly coated on the transparent substrate (glass plate) to ensure that the surface was smooth and free of bubbles, and the coating was cured. The coated substrate was placed in the sample chamber, ensuring that the surface of the film was perpendicular to the light path. A test wavelength range of 380–780 nm was selected. Starting the test, the instrument automatically measured the transmission light intensity of the UV topcoat film at different wavelengths.

Glossiness test of UV topcoat film: The glossiness of the UV topcoat film was tested according to GB/T 4893.6-2013 [43]. A gloss meter was used to measure the glossiness of the UV topcoat film when exposed to light sources at different angles. The gloss meter HG268 was used to measure the glossiness, and the inside of the instrument was illuminated by an LED light source, which was emitted through the bottom test window. The light source for glossiness testing had an incidence angle of 60°. When the gloss meter was used to test the surface glossiness of the UV topcoat film, the measurement window at the bottom of the instrument with the UV topcoat film was covered, the measurement button was clicked, and then the data was recorded. The surface glossiness of the UV topcoat film with microcapsules was recorded as *G*_1_, and the surface glossiness of the UV topcoat film without microcapsules was recorded as *G*_0_. The formula for calculating the gloss loss rate *G* is shown in Formula (5).

*G* = [(*G*_0_ − *G*_1_)/*G*_0_] × 100% (5)

Cold liquid resistance test of UV topcoat film: According to GB/T 4893.1-2021 [44], the UV topcoat film was tested and evaluated, and deionized water was selected as the test liquid. A circular filter paper piece with a specification of 25 ± 2 mm was immersed in the deionized water for 30 s, and then picked up with tweezers and placed on the surface of the UV topcoat film, then covered with a petri dish. After being placed in an environment with a temperature of 23 °C and a humidity of 50%RH for 24 h, the UV topcoat film was taken out and the surface was dried. Under light conditions, the changes in the gloss of the UV topcoat film were checked, including whether there were any defects such as bubbles or cracks. The cold liquid resistance grade of the UV topcoat film was evaluated in accordance with GB/T 4893.1-2021.

Aging test of UV topcoat film: According to GB/T 1766-2008 [45], the UV topcoat film was subjected to an aging resistance test, whereby the UV yellowing resistance test chamber WJ-UV-150 was used. All surfaces of the sample except the test area were sealed with an edge-sealing agent composed of rosin and paraffin. The samples were then placed in the UV yellowing resistance test chamber. The conditions of the UV yellowing resistance test chamber were set to a temperature of 50 °C, a humidity of 99%RH, and an irradiation intensity of 0.55 W/m^2^. A radiation cycle consisted of 4 h of heating at 60 °C followed by 4 h of condensation at 50 °C. After 48 h, the samples were removed, and their gloss and chromaticity values were measured. The gloss loss rate was calculated using Formula (5). The values of the blank UV topcoat film were recorded as *L*_1_, *a*_1_, and *b*_1_*,* and the values of the UV topcoat film containing microcapsules were recorded as *L*_2_, *a*_2_, and *b*_2_. Formula (6) was used to calculate the chromaticity values Δ*E* of the UV topcoat film. Among them, Δ*L = L*_2_ *− L*_1_, Δ*a = a*_2_ *− a*_1_, and Δ*b = b*_2_ *− b*_1_.

Δ*E* = [(Δ*L*)^2^ + (Δ*a*)^2^ + (Δ*b*)^2^]^1/2^ (6)

Each of the above tests was performed four times, and the results were averaged.

## 3. Results and Discussion

### 3.1. Microcapsules Testing

#### 3.1.1. Yield and Coverage Rate Test

The yield and coverage rate of microcapsules have important effects on the performance of the microcapsules. There is less core material content in microcapsules with a low coating rate, and there is less of the self-repairing agent in the wall material. Therefore, their self-repairing performance will be degraded, and low-yield microcapsules will increase production costs. Therefore, it was necessary to determine the preparation process of the microcapsule samples with a better coverage rate and yield through statistical analysis of the results of the four-factor and three-level orthogonal tests. In the orthogonal tests, the coverage rate of microcapsules #1 was the highest, and the coverage rate reached 54.00%, followed by microcapsules #5, which had a coverage rate of 42%. Microcapsules #3 had the lowest coverage rate of 20.00%. The analysis results of the influence of each factor level on the microcapsules’ coverage rates are shown in Table 6. The K value in the table was used to represent the relationship between each factor and the result. k was used to distinguish the level that had the greatest impact. R was the range, indicating the influence of various factors on the coverage rate. By comparing the range values of the four factors, D > B > A > C was obtained. The results showed that the drying temperature was the most important factor affecting the sample coverage rate, followed by the pH value and core–wall mass ratio. The mass ratio of the wall material of chitosan and Arabic gum had little effect on the result. Comparing the K-value of each column, the optimal levels of each factor were A1B2C1D1. A1 means the level 1 of factor A in the orthogonal experiment. B2 means the level 2 of factor B. C1 means the level 1 of factor C. D1 means the level 1 of factor D. A1B2C1D1 means that the preparation methods of microcapsules with a better coverage rate were a core–wall ratio of 0.5:1.0, pH value of 3.5, feeding mass ratio of chitosan and Arabic gum of 1:4, and drying temperature of 150 °C. This is because when the inlet temperature of spray drying is too high, it may cause the solidification of the core Tung oil, while the pH value during complex coacervation is related to the degree of condensation between chitosan and Arabic gum. At the same time, a high core–wall mass ratio may prevent the wall material from fully covering the core material.

Yield was another indicator for measuring the quality of microcapsules, calculated by the ratio of the quality of microcapsules collected after preparation to the quality of the raw materials used. The relationships between the levels of various factors and the yield are shown in Table 7. The highest yield of microcapsules #3 in the orthogonal experiments was 43.14%, followed by microcapsules #6, with a yield of 33.89%. The lowest yield was 23.07% for microcapsules #7. The impacts of various influencing factors on the yield of microcapsules were as follows: D > A > B > C. The biggest influence factor was the inlet temperature during spray drying, followed by the core–wall mass ratio and pH value, and the smallest influence factor on the yield result was the ratio of the two wall materials. Under the conditions of these four-factor three-level orthogonal experiments, the factor levels required for the preparation process of microcapsule samples that could achieve high yields were A1B3C1D3. A1B3C1D3 means that the preparation process included a core–wall mass ratio of 0.5:1.0, a pH value of 4 for the complex coagulation reaction, a mass ratio of the wall material chitosan to Arabic gum of 1.0:4.0, and a drying temperature of 190 °C, the highest yield of microcapsule samples could be obtained.

The results of the four-factor and three-level orthogonal experiments designed with the core–wall mass ratio, the pH value of the complex coagulation reaction, the ratio of the two wall materials, and the temperature during spray drying as factors showed that the drying temperature during the preparation process had a great influence on the coverage rate and yield of microcapsules. Based on the conditions given by the orthogonal experiments, the preparation process required to obtain the sample with the best coverage rate included a core–wall mass ratio of 0.5:1.0, a pH value of 3.5, a chitosan–Arabic gum mass ratio of 1.0:4.0, and a drying temperature of 150 °C. The preparation process for obtaining the sample with the best yield included a core–wall mass ratio of 0.5:1.0, a pH value of 4 for the complex coagulation reaction, a mass ratio of the wall material chitosan to Arabic gum of 1.0:4.0, and a drying temperature of 190 °C. Due to the significant impact of factor D on the encapsulation rate and yield of microcapsules, a single-factor experiment was designed with factor D as the variable. Due to the preparation conditions of a core–wall mass ratio of 0.5:1.0 and a mass ratio of chitosan to Arabic gum of 1.0:4.0, microcapsules were prepared at optimal levels in terms of encapsulation efficiency and yield. Therefore, in the single-factor experiment, the core–wall mass ratio of 0.5:1.0 and mass ratio of chitosan to Arabic gum of 1.0:4.0 were adopted as the test invariants. The higher the coverage rate, the higher the content of core material in the microcapsules, and these core materials were used as self-repairing agents to determine the self-repairing performance of microcapsules. In order to achieve a higher coverage rate, factor B adopted the level B2, that was more conducive to obtaining a higher coverage rate. The single-factor experiment was designed with A1B2C1 as the invariant conditions and factor D as the variable to further determine the preparation conditions of the microcapsules.

The single-factor test was designed with a core–wall mass ratio of 0.5:1.0, pH value of 3.5, mass ratio of chitosan to Arabic gum of 1.0:4.0, and the test variable, the spray drying temperature. The test results are shown in Table 8. The microcapsule samples were prepared using the drying temperature as a variable, and the yield of the samples showed a trend of first increasing and then decreasing with increasing temperature. Microcapsules #12, prepared at a drying temperature of 130 °C, achieved the highest yield in the single-factor experiment, with a yield of 32.85%. The coverage rate of each microcapsule sample also showed the same trend with increasing temperature. Microcapsules #11, prepared at a drying temperature of 120 °C, achieved the highest coverage rate of 33.25% in the single-factor experiment. Based on the yields and coverage rates of all microcapsules in the single-factor test, the best sample in the test was microcapsules #12, with a core–wall ratio of 0.5:1.0, pH value of 3.5, mass ratio of chitosan to Arabic gum of 1.0:4.0, and spray drying temperature of 130 °C.

#### 3.1.2. Morphology Testing of Microcapsules

**Figure 1:**
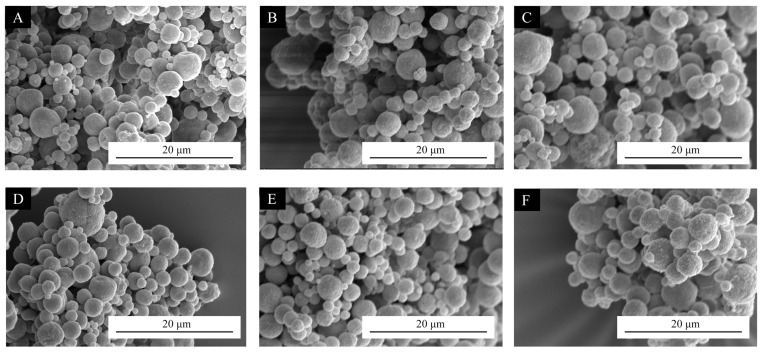
Microcapsules under SEM: (**A**) microcapsules #10, (**B**) microcapsules #11, (**C**) microcapsules #12, (**D**) microcapsules #13, (**E**) microcapsules #14, and (**F**) microcapsules #15.

The microstructures of several microcapsule samples prepared in the single-factor experiment are shown in Figure 1. As shown in Figure 1, microcapsules #10–#12, prepared at drying temperatures of 110 °C, 120 °C, and 130 °C, showed more pronounced adhesion between the spheres of microcapsules compared to microcapsules #13–#15, prepared at 140 °C, 150 °C, and 160 °C. This indicates that microcapsules prepared at higher drying temperatures can obtain more complete spheres compared to those prepared at lower drying temperatures. With the increase of the spray drying temperature, the surface morphology of microcapsules gradually became smooth, and the spherical microcapsules were more independent [46].

**Figure 2:**
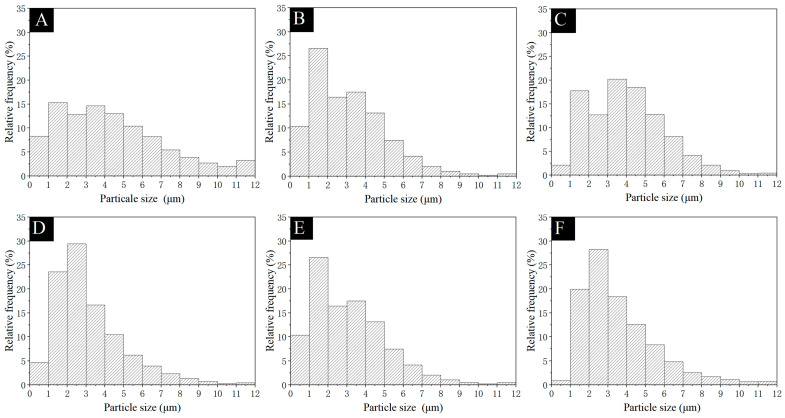
Particle size: (**A**) microcapsules #10, (**B**) microcapsules #11, (**C**) microcapsules #12, (**D**) microcapsules #13, (**E**) microcapsules #14, and (**F**) microcapsules #15.

The histogram of the microcapsule particle size distribution is shown in Figure 2. The particle sizes of the several microcapsules prepared were similar, mostly ranging from 1 μm to 5 μm. Because the particle sizes of microcapsules were mainly controlled by the rotational speed of the magnetic stirrer during preparation, the larger the rotational speed, the smaller the microcapsule particle size obtained [47]. All preparation processes were the same except for the final drying stage; therefore, the microcapsule samples obtained in the single-factor experiment have a certain degree of similarity.

#### 3.1.3. Microcapsule Chemical Composition Testing

**Figure 3:**
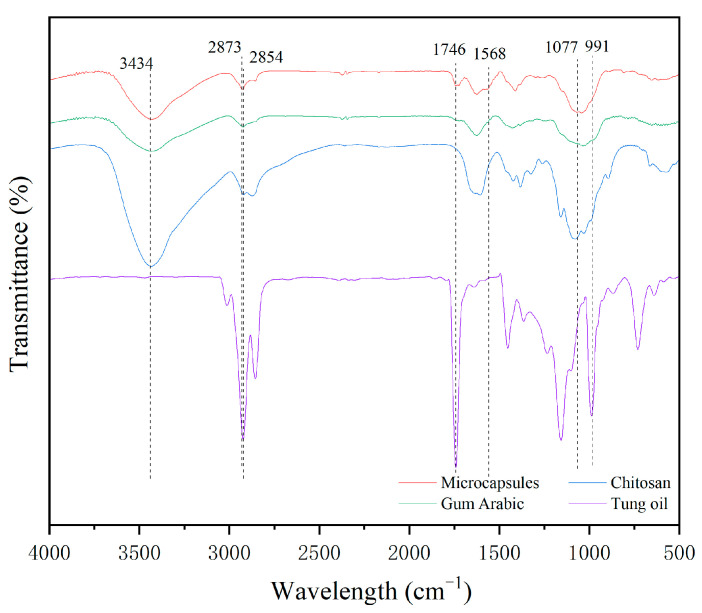
The infrared spectra of the microcapsules and their materials.

The infrared spectra of the Tung oil microcapsules coated with chitosan–Arabic gum are shown in Figure 3. The C-H stretching vibration band at 2854 cm^−1^, the C=O stretching vibration band at 1746 cm^−1^, and the conjugated double bond bending vibration band at 991 cm^−1^ are the characteristic peaks of Tung oil [48], indicating the presence of Tung oil in the sample. The vibration absorption band of -OH at 3434 cm^−1^, the stretching vibration band of -CH at 2873 cm^−1^, and the absorption band of C-O-C at 1077 cm^−1^ are the characteristic bands of chitosan [49], indicating the presence of chitosan in the sample. Due to the polysaccharide nature of Arabic gum, the band around 3500 cm^−1^ is a stretching vibration band generated by -OH. The microcapsules contain characteristic bands of the core and wall materials, and the band at 1568 cm^−1^ is the electrostatic interaction between the -COOH of Arabic gum in the wall material and the -NH of chitosan [50]. This indicates that the core material, Tung oil, was encapsulated in the wall material, chitosan–Arabic gum, and the chitosan–Arabic gum-coated Tung oil microcapsules were successfully prepared.

### 3.2. UV Topcoat Testing

#### 3.2.1. Surface Morphology of UV Topcoat

**Figure 4:**
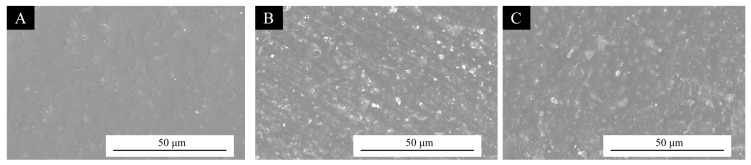
Surface morphology of UV topcoat film under SEM: (**A**) blank UV topcoat, (**B**) UV topcoat with 5% microcapsules #11 added, and (**C**) UV topcoat with 5% microcapsules #12 added.

The surface morphology images of the blank UV topcoat, UV topcoat with 5% microcapsules #11 added, and UV topcoat with 5% microcapsules #12 added under SEM are shown in Figure 4. The surface of the UV topcoat film without microcapsules was relatively flat, but after adding microcapsules, the uneven distribution of microcapsules made the surface of the UV topcoat film uneven. The surface morphologies of the UV topcoat film after adding the two types of microcapsules have a certain similarity, which is due to the fact that both types of microcapsules were added to the UV topcoat film at a rate of 5% and have similar morphology.

#### 3.2.2. Chemical Composition Testing of UV Topcoat

**Figure 5:**
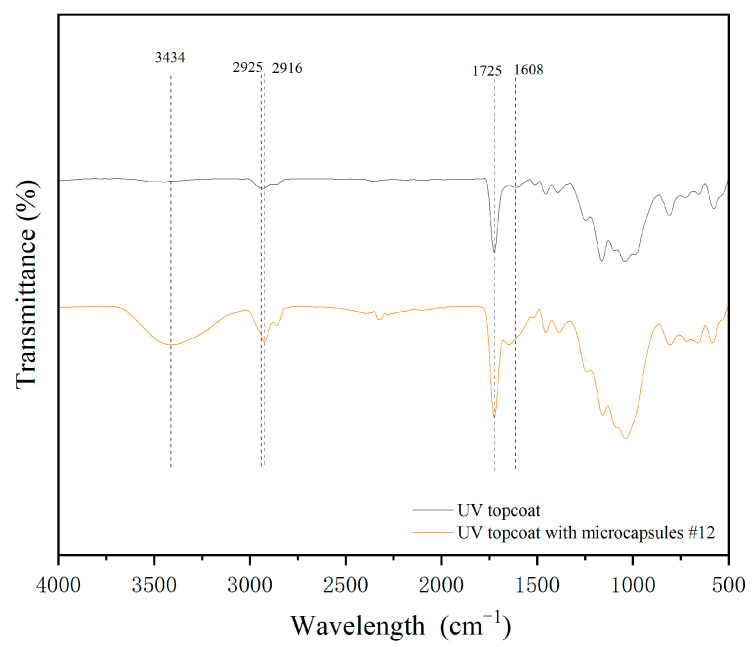
Infrared spectra of microcapsules and UV topcoat film.

The infrared spectra of the UV topcoat film without microcapsules and the UV topcoat film with microcapsules #11 added are shown in Figure 5. The main components of the UV topcoat were polyurethane acrylic resin, propylene glycol diacrylate, and hexanediol diacrylate. The C=C stretching vibration band at 1608 cm^−1^, the C=O vibration band at 1725 cm^−1^, and the C-H stretching vibration band at 2925 cm^−1^ are common characteristic bands of the main components in UV topcoats [30]. The most prominent broad band at 3434 cm^−1^ in the infrared spectrum of the UV topcoat with microcapsules #11 comes from chitosan in the microcapsule wall material. In the curve of adding microcapsules, characteristic bands of the UV topcoat can be observed simultaneously, indicating that the addition of microcapsules did not react chemically with the components in the UV topcoat.

#### 3.2.3. Self-Repairing Performance Test of UV Topcoat

**Figure 6:**
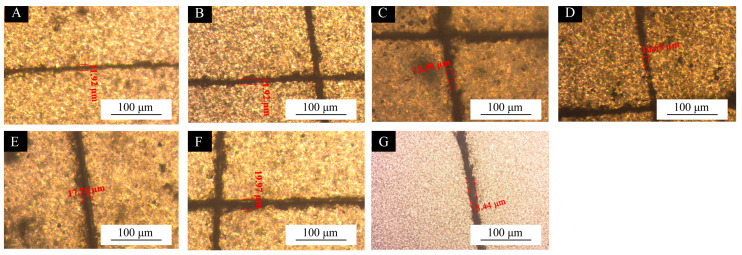
Scratch width before self-repairing: (**A**) UV topcoat with microcapsules #10, (**B**) UV topcoat with microcapsules #11, (**C**) UV topcoat with microcapsules #12, (**D**) UV topcoat with microcapsules #13, (**E**) UV topcoat with microcapsules #14, (**F**) UV topcoat with microcapsules #15, and (**G**) UV topcoat.

**Figure 7:**
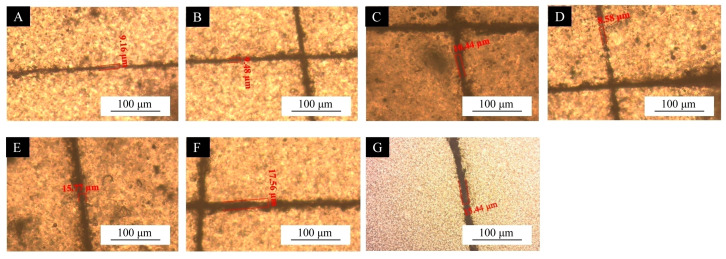
Scratch width after self-repairing: (**A**) UV topcoat with microcapsules #10, (**B**) UV topcoat with microcapsules #11, (**C**) UV topcoat with microcapsules #12, (**D**) UV topcoat with microcapsules #13, (**E**) UV topcoat with microcapsules #14, (**F**) UV topcoat with microcapsules #15, and (**G**) UV topcoat.

The surface self-repairing performance of the UV coatings with different microcapsule samples is shown in Figure 6 and Figure 7 and Table 9. Compared with the blank UV topcoats, the UV topcoats containing microcapsules exhibited reparative properties for scratches. This indicates that the self-repairing performance of the coating on scratches was not significant when using UV topcoats without the addition of self-repairing microcapsules. The addition of self-healing microcapsules enables the coating to achieve scratch self-repairing performance. Scratch tests were conducted on UV topcoat films with different microcapsules added. Among them, coatings with microcapsules #10 showed the best self-repairing performance, achieving a 23.15% self-repair of scratches after 48 h. The coating of microcapsules #11 also showed a better self-repairing effect on scratches; the self-repairing rate was 20.47% after 48 h. Although the coatings of microcapsules #14 and #15 also showed some self-repairing effects, their self-repairing ability was weaker compared to other samples, at only 11.30% and 12.07%, respectively. From this, it can be seen that several Tung oil microcapsules coated with chitosan–Arabic gum prepared in the single-factor experiment can endow the UV topcoat film with a self-repairing function. When the coating is damaged, the wall material of the microcapsules added into the UV paint breaks along with the scratch, and the Tung oil in the core material flows out and reacts with oxygen in the air to solidify, thus achieving a certain degree of closure of the scratch.

**Figure 8:**
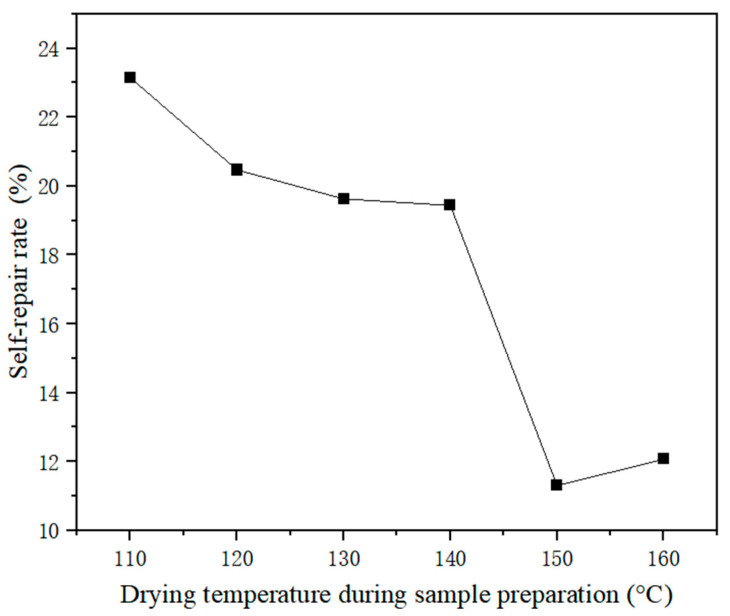
The effect of drying temperature on the self-repairing rate of microcapsules.

As shown in Figure 8, when the microcapsules #10, prepared at a spray drying temperature of 110 °C, were added to the UV topcoat with 5% content, the self-repairing rate of the UV topcoat film was 23.15%. Microcapsules #11, prepared at a temperature of 120 °C, can also achieve a self-repairing rate of 20.47% when added to the coating at a content of 5%. Microcapsules #12, prepared at a temperature of 130 °C, can also achieve a self-repairing rate of 19.63% when added to the coating at a content of 5%. However, as the temperature continued to rise, the self-repairing performance of the produced microcapsule samples gradually decreased. The spray drying temperature was set as a variable in the design of the single-factor test. The results of the single-factor test showed that the coating rate of microcapsules decreased with the increase of temperature. Therefore, it was concluded that the increase of the temperature during spray drying would lead to the decrease of the coating rate of microcapsules. The low coating rate of the microcapsules means that there was less self-repairing agent in them, which leads to the low self-repairing rate of the UV topcoat film added with these microcapsules.

#### 3.2.4. Mechanical Testing of UV Topcoat

A roughness meter is commonly used to measure the roughness of UV topcoat film surfaces. A lower value indicates a smoother UV topcoat film surface, while a higher value indicates a more uneven surface. The surface roughness of the UV topcoat films with different types of microcapsules added are shown in Table 10. Each sample was added to the UV topcoat at a content of 5%. The highest surface roughness was observed in the UV topcoat film with added microcapsules #13, with a roughness of 0.87 μm. The UV topcoat without microcapsules had the lowest roughness, with a roughness of 0.31 μm. The results showed that the addition of Tung oil microcapsules coated with chitosan–Arabic gum increased the surface roughness of the UV topcoat. This is because when solid microcapsules are added to the liquid state UV topcoat film, solid particles agglomerate in the liquid and cannot be uniformly dispersed in the liquid. Therefore, UV topcoats containing microcapsules have uneven surfaces after curing, resulting in an increase in surface roughness of the UV topcoat.

Table 10 shows the fracture elongation of UV topcoat films with different microcapsules added. The elongation at break of the UV topcoat film is usually used to measure the ductility of the UV topcoat film, and UV topcoat films with better ductility have higher elongation at break. The fracture elongation of the UV topcoat film without microcapsules added was 4.43%, while the fracture elongation of the UV topcoat film with microcapsules added could be improved to some extent. This is because the addition of microcapsules improves the strength of the UV topcoat film, but due to the high solid content of the UV topcoat, the hardness and toughness of the cured UV topcoat film are relatively high, resulting in a lower breaking elongation.

#### 3.2.5. Optical Testing of UV Topcoat

**Figure 9:**
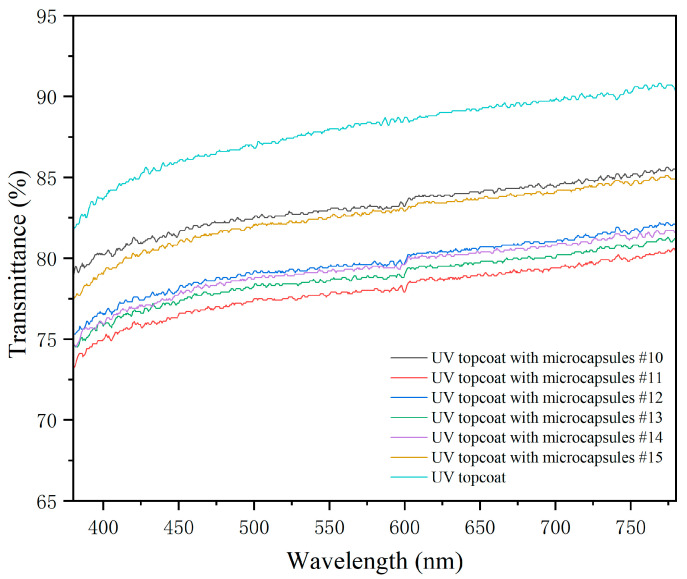
Transmittance rates of UV topcoat films.

As shown in Figure 9 and Table 11, the transmittance of the UV topcoat film with different microcapsules added and without microcapsules were presented. The visible light transmittance of the UV topcoat film can reflect the transparency of the UV topcoat film, and the higher the transmittance, the more transparent the UV topcoat film. The visible light transmittance of UV topcoat without microcapsules was the highest, reaching 87.90%. Adding microcapsules to UV topcoats can lead to a decrease in the transmittance of the UV topcoat film. UV topcoats with microcapsules #10 had a higher visible light transmittance of 83.13%. The visible light transmittance of the UV topcoat film with microcapsules #11 added was the lowest, at 77.96%. The reason why the UV topcoat film with the addition of #10 microcapsules has a stronger light transmittance compared to other UV topcoat films is that the particle sizes of #10 microcapsules are more uniform than the other samples, and thus have a smaller impact on the UV topcoat film. The visible light transmittance of the UV topcoat film after adding microcapsules decreases to varying degrees. This is due to the increased surface roughness of the UV topcoat film after adding solid particles, which exacerbates the diffuse reflection phenomenon of the UV topcoat film and leads to a decrease in transmittance.

The glossiness of the UV topcoat film surface is an important indicator for measuring the reflective ability of the UV topcoat film surface to light, and is usually used to evaluate the appearance quality of the UV topcoat film. The higher the glossiness value, the better the glossiness of the UV topcoat film surface. The sample numbered 0 was a UV topcoat film without microcapsules. Table 12 shows the glossiness of the UV topcoat surfaces when different microcapsules were added. The glossiness of the blank UV topcoat film without microcapsules was the highest, reaching 49.30 GU at incident angle of 60°. The glossiness was reduced to varying degrees after adding microcapsules. The lower the gloss loss rate of the UV topcoat film, the higher the gloss of the UV topcoat film. The lowest gloss loss rate of the UV topcoat film, with the addition of microcapsules #11, was 10.95%. The gloss loss rate of the UV topcoat film with the addition of microcapsules #15 was relatively high, at 23.73%. This indicates that the UV topcoat film with the addition of microcapsules #11 had good glossiness, while the UV topcoat film with the addition of microcapsules #15 had poor glossiness. Compared with the UV topcoat film without microcapsules, due to the different spray drying temperatures during sample preparation, the opacity of the UV topcoat film surface with different microcapsules increased first and then decreased. Microcapsules #11, prepared at the spray drying temperature of 120 °C, were added to the UV topcoat, which could keep the UV topcoat film with good gloss. When the incident angle of the light source was 60°, the glossiness could reach 43.90 GU.

#### 3.2.6. Cold Liquid Resistance Testing of UV Topcoat

**Figure 10:**
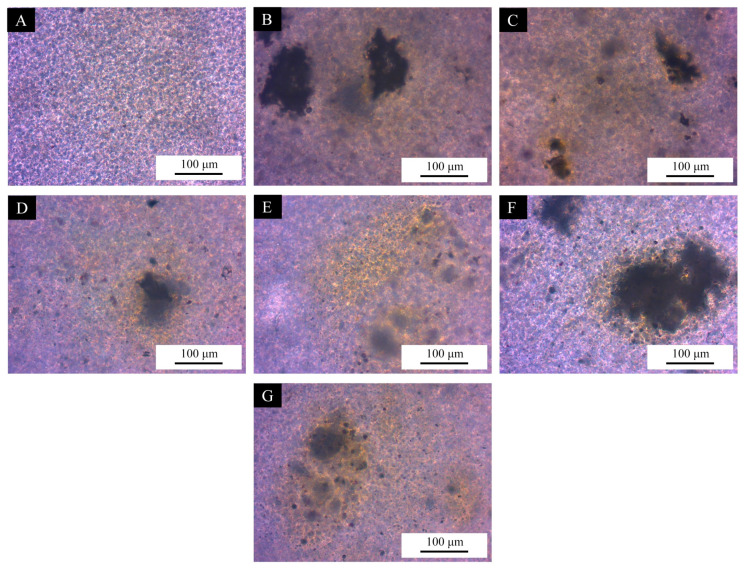
Microscopic morphology of the UV topcoat film after the cold liquid resistance test: (**A**) UV topcoat with microcapsules #10, (**B**) UV topcoat with microcapsules #11, (**C**) UV topcoat with microcapsules #12, (**D**) UV topcoat with microcapsules #13, (**E**) UV topcoat with microcapsules #14, (**F**) UV topcoat with microcapsules #15, and (**G**) UV topcoat.

When conducting the cold liquid resistance test of the UV topcoat film, deionized water was selected as the test liquid. According to GB/T 4893.1-2021, when the test area and the adjacent area could not be distinguished, the cold liquid resistance grade of the UV topcoat film was recorded as grade 1. When the test area and the adjacent area could be distinguished but no cracking or swelling occurred on the surface, it was recorded as grade 2. Figure 10 presents the microscopic morphology of the UV topcoat film after cold liquid resistance testing, observed under an optical microscope. The coating exhibited no cracking or swelling; however, yellowing appeared around the aggregated microcapsules. This phenomenon may be attributed to water absorption-induced wall material swelling during immersion in deionized water, which increased the pore size and facilitated outward diffusion of the Tung oil components. Table 13 shows the gloss changes of the UV topcoat films with different microcapsules before and after the cold liquid resistance test, as well as the evaluation of the test results of each UV topcoat film. The gloss of the UV topcoat without microcapsules changed little compared to before the test, and no bubbles or cracking occurred. The glosses of the UV topcoats with microcapsules all decreased, fluctuating between 38.80 and 22.30 GU. This is because the UV topcoats were composed of cross-linked and dense hydrophobic resins, which have strong resistance to deionized water and a low water absorption rate, so the gloss change before and after the test was small. The wall material of the microcapsules added to the UV topcoat was composed of chitosan and Arabic gum. Both chitosan and Arabic gum contain a large amount of —OH [51], which has a certain degree of water absorption. Therefore, during immersion in deionized water, the shells of the microcapsules swell due to water absorption, causing the formation of microscopic concave convex structures, thereby reducing glossiness.

#### 3.2.7. Aging Testing of UV Topcoat

**Figure 11:**
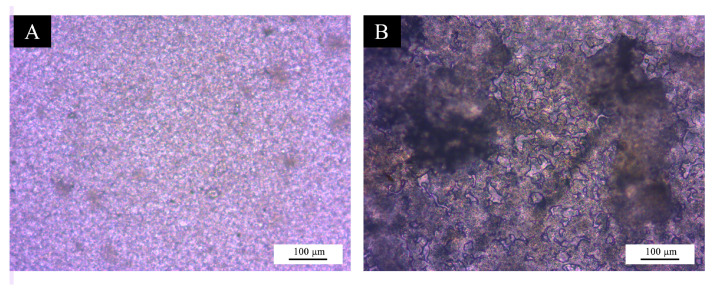
Microscopic morphology of the UV topcoat film after the aging test: (**A**) UV topcoat, and (**B**) UV topcoat with microcapsules #11.

The UV topcoat with microcapsules #11, which exhibited relatively comprehensive test performance, was selected along with the blank UV topcoat for aging tests. The test samples were placed in a UV yellowing resistance test chamber, and the test was terminated after 48 h. The glossiness and Δ*E* of the UV topcoat films were measured. The glossiness and color difference values before and after aging are shown in Table 14. The gloss of the blank UV topcoat film decreased from 49.30 GU to 41.00 GU, while that of the UV topcoat with microcapsules #11 decreased from 43.90 GU to 26.30 GU. Compared to the microcapsule-free paint, the glossiness change of the microcapsule-containing paint was more significant before and after aging, with the gloss loss rate increasing from 10.95% to 35.85%. Before aging, the Δ*E* between the UV topcoat with microcapsules #11 and the blank UV topcoat was 5.83, which increased to 13.32 after aging. The addition of microcapsules reduces the aging resistance of the UV topcoat. The UV topcoat showed no obvious changes after the aging test, whereas the UV topcoat with microcapsules #11 exhibited partial delamination from the substrate. The microscopic morphology observed under an optical microscope is shown in Figure 11. The blank UV topcoat film remained largely unchanged, while the texture of the UV topcoat with microcapsules #11 displayed significant alterations. This was attributed to the high humidity inside the test chamber, where the hygroscopic swelling behavior of the Tung oil microcapsules coated with chitosan–Arabic gum led to interfacial delamination failure between the UV topcoat film and substrate. Additionally, water absorption-induced pore enlargement in the chitosan–Arabic gum wall material facilitated the outward diffusion of the Tung oil components, resulting in an increased color difference of the UV topcoat film.

## 4. Conclusions

The complex coagulation method was used to prepare Tung oil microcapsules coated with chitosan–Arabic gum. Four-factor and three-level orthogonal experiments were designed with the core–wall mass ratio, pH value in the complex coagulation reaction, ratio of the two wall materials, and temperature of spray drying as variables. The experiment showed that the temperature of spray drying was the biggest factor affecting the preparation of microcapsules. A single-factor experiment was designed with the temperature of spray drying as a variable to select the appropriate temperature for preparing Tung oil microcapsules coated with chitosan–Arabic gum. The microcapsules prepared under the conditions of a 0.5:1.0 core–wall ratio, 3.5 pH value, 1.0:4.0 mass ratio of chitosan to Arabic gum, and 130 °C spray drying temperature have a high yield and coating rate, which were 32.85% and 33.20%, respectively. The microcapsules obtained from the single-factor experiment were added to the UV topcoat at a content of 5.0%. With the increase of the spray drying temperature, the roughness of the UV topcoat film first increased and then decreased. The elongation at break also showed a trend of first increasing and then decreasing. The visible light transmission of the UV topcoat film first decreased and then increased, and the glossiness showed a trend of first increasing and then decreasing. With the increase of the spray drying temperature during the preparation of the microcapsules, the self-repairing rate of the prepared the UV topcoat film gradually decreased, and the self-repairing rate of the UV topcoat film added with microcapsules #10 was the highest, at 23.15%. However, the addition of microcapsules reduced the cold liquid resistance and aging resistance of the UV topcoat film. When microcapsules #11 were added to the UV topcoat at a rate of 5.0%, the UV topcoat film could achieve excellent comprehensive performance in terms of self-repairing, mechanical, and optical properties. The self-repairing rate of the UV topcoat film with microcapsules #11 was 20.47%, the visible light transmittance was 77.96%, the gloss loss rate was 10.95%, the roughness was 0.79 μm, and the breaking elongation was 5.04%. Tung oil microcapsules coated with chitosan–Arabic gum added to UV topcoats can enable the UV topcoat film to achieve self-repairing performance. The preparation of Tung oil microcapsules coated with chitosan–Arabic gum proves the feasibility of using natural materials as raw materials to prepare self-repairing microcapsules.

## Figures and Tables

**Table 1:** List of materials used in the test.

Materials	Molecular Formula	CAS	Producer
Chitosan	(C_6_H_11_NO_4_)_n_	9012-76-4	China National Pharmaceutical Group Chemical Reagent Co., Ltd., Shanghai, China
Arabic gum	C_12_H_36_	9000-01-5	Tianjin Zhonglian Chemical Reagent Co., Ltd., Tianjin, China
Acetic acid	C_2_H_4_O_2_	64-19-7	China National Pharmaceutical Group Chemical Reagent Co., Ltd., Shanghai, China
Tung oil	C_65_H_100_O_14_	-	Shanghai Shenmeng Home Furnishings Co., Ltd., Shanghai, China
Tannic acid	C_76_H_52_O_46_	1401-55-4	Tianjin Zhonglian Chemical Reagent Co., Ltd., Tianjin, China

**Table 2:** List of equipment used in the experiment.

Equipment	Model	Manufacturer
Heat collecting magnetic stirrer	DF-101Z	Shanghai Yixin Scientific Instrument Co., Ltd., Shanghai, China
Ultrasonic emulsifier disperser	BILONG-500	Shanghai Bilon Instrument Co., Ltd., Shanghai, China
Spray dryer	JA-PWGZ100	Shenyang Jingao Instrument Technology Co., Ltd., Shenyang, China
Circulating water vacuum pump	SHZ-D	Shanghai Simite Instrument Equipment Co., Ltd., Shanghai, China
UV curing machine	620#	Huzhou Tongxu Machinery Equipment Co., Ltd., Huzhou, China
Roughness tester	J8-4C	Shanghai Taiming Optical Instrument Co., Ltd., Shanghai, China
Gloss meter	HG268	Shenzhen 3nh Technology Co., Ltd., Shenzhen, China
Universal mechanics testing machine	AGS-X	Shimazu Manufacturing Co., Ltd., Kyoto, Japan
Ultraviolet spectrophotometer	U-3900	Hitachi Instruments (Suzhou) Co., Ltd., Suzhou, China
Scanning electron microscope (SEM)	QUANTA-200	FEI Company, Hillsboro, OR, USA
Infrared spectrometer	VERTEX 80V	Germany BRUKER Co., Ltd., Karlsruhe, Germany
UV yellowing resistance test chamber	WJ-UV-150	Hangzhou Wujia machine Co., Ltd., Hangzhou, China

**Table 3:** Orthogonal experimental design.

Levels	Factor A Core–Wall Mass Ratio	Factor B pH	Factor C The Ratio of Two Wall Materials	Factor D The Temperature During Spray Drying (°C)
1	0.5:1.0	3.0	1.0:4.0	150
2	0.8:1.0	3.5	1.0:5.0	170
3	1.0:1.0	4.0	1.0:6.0	190

**Table 4:** Orthogonal experimental schedule.

Sample (#)	Factor A Core–Wall Mass Ratio	Factor B pH	Factor C The Ratio of Two Wall Materials	Factor D The Temperature During Spray Drying (°C)
1	0.5:1.0	3.0	1.0:4.0	150
2	0.5:1.0	3.5	1.0:5.0	170
3	0.5:1.0	4.0	1.0:6.0	190
4	0.8:1.0	3.0	1.0:5.0	190
5	0.8:1.0	3.5	1.0:6.0	150
6	0.8:1.0	4.0	1.0:4.0	170
7	1.0:1.0	3.0	1.0:6.0	170
8	1.0:1.0	3.5	1.0:4.0	190
9	1.0:1.0	4.0	1.0:5.0	150

**Table 5:** List of experimental materials.

Text	Sample (#)	Arabic Gum (g)	Deionized Water (mL)	Chitosan (mL)	1% Acetic Acid (g)	Tung Oil (g)	Tannic Acid (g)	Deionized Water (mL)
Orthogonal experiments	1	3.200	76.800	0.800	79.200	2.000	0.020	9.980
	2	4.000	76.000	0.800	79.200	2.400	0.020	9.980
	3	4.800	75.200	0.800	79.200	2.800	0.020	9.980
	4	4.000	76.000	0.800	79.200	3.840	0.020	9.980
	5	4.800	75.200	0.800	79.200	4.480	0.020	9.980
	6	3.200	76.800	0.800	79.200	3.200	0.020	9.980
	7	4.800	75.200	0.800	79.200	5.600	0.020	9.980
	8	3.200	76.800	0.800	79.200	4.000	0.020	9.980
	9	4.000	76.000	0.800	79.200	4.800	0.020	9.980
Single-factor experiment	10	3.200	76.800	0.800	79.200	2.000	0.020	9.980
	11	3.200	76.800	0.800	79.200	2.000	0.020	9.980
	12	3.200	76.800	0.800	79.200	2.000	0.020	9.980
	13	3.200	76.800	0.800	79.200	2.000	0.020	9.980
	14	3.200	76.800	0.800	79.200	2.000	0.020	9.980
	15	3.200	76.800	0.800	79.200	2.000	0.020	9.980

**Table 6:** Analysis of microcapsules’ coverage rates in orthogonal tests.

Sample (#)	Factor A Core–Wall Mass Ratio	Factor B pH	Factor C The Ratio of Two Wall Materials	Factor D The Temperature During Spray Drying	*C* (%)
1	0.5:1.0	3.0	1.0:4.0	150	54.00
2	0.5:1.0	3.5	1.0:5.0	170	36.00
3	0.5:1.0	4.0	1.0:6.0	190	20.00
4	0.8:1.0	3.0	1.0:5.0	190	22.00
5	0.8:1.0	3.5	1.0:6.0	150	42.00
6	0.8:1.0	4.0	1.0:4.0	170	24.00
7	1.0:1.0	3.0	1.0:6.0	170	36.00
8	1.0:1.0	3.5	1.0:4.0	190	35.00
9	1.0:1.0	4.0	1.0:5.0	150	38.00
K_1_	110.00	112.00	113.00	134.00	
K_2_	88.00	113.00	96.00	96.00	
K_3_	109.00	82.00	98.00	77.00	
k_1_	36.67	37.33	37.67	44.67	
k_2_	29.33	37.67	32.00	32.00	
k_3_	36.33	27.33	32.67	25.67	
R	7.33	10.33	5.67	19.00	
Order of influencing factors	D > B > A > C				
Optimal level	A1	B2	C1	D1	
Recommended preparation process	A1B2C1D1				

**Table 7:** Analysis of microcapsule yield rates in the orthogonal experiments.

Sample (#)	Factor A Core–Wall Mass Ratio	Factor B pH	Factor C The Ratio of Two Wall Materials	Factor D The Temperature During Spray Drying	*Y* (%)
1	0.5:1.0	3.0	1.0:4.0	150	26.80
2	0.5:1.0	3.5	1.0:5.0	170	30.89
3	0.5:1.0	4.0	1.0:6.0	190	43.14
4	0.8:1.0	3.0	1.0:5.0	190	32.13
5	0.8:1.0	3.5	1.0:6.0	150	25.79
6	0.8:1.0	4.0	1.0:4.0	170	33.89
7	1.0:1.0	3.0	1.0:6.0	170	23.07
8	1.0:1.0	3.5	1.0:4.0	190	31.40
9	1.0:1.0	4.0	1.0:5.0	150	25.50
K_1_	100.83	82.00	92.09	78.09	
K_2_	91.81	88.08	88.52	87.85	
K_3_	79.97	102.53	92.00	106.67	
k_1_	33.61	27.33	30.70	26.03	
k_2_	30.63	29.36	29.51	29.28	
k_3_	26.67	34.18	30.67	35.56	
R	6.95	6.84	1.19	9.53	
Order of influencing factors	D > A > B > C				
Optimal level	A1	B3	C1	D3	
Recommended preparation process	A1B3C1D3				

**Table 8:** Microcapsules’ yield rates and encapsulation rates in the single-factor test.

Sample (#)	Factor D The Temperature During Spray Drying (°C)	*Y* (%)	*C* (%)
10	110	29.65	28.75
11	120	29.79	33.25
12	130	32.85	33.20
13	140	31.25	32.40
14	150	30.05	31.25
15	160	28.86	25.75

**Table 9:** Self-repairing rate of each sample in the scratch test.

Microcapsules (#)	Temperature During Spray Drying (°C)	Scratch Width Before Repair (μm)	Scratch Width After Repair (μm)	Self-Repairing Rate (%)
10	110	11.92	9.16	23.15
11	120	11.92	9.48	20.47
12	130	12.99	10.44	19.63
13	140	10.65	8.58	19.44
14	150	17.78	15.77	11.30
15	160	19.97	17.56	12.07

**Table 10:** Surface roughness and breaking elongation of UV topcoat films with different microcapsules added.

Microcapsules (#)	Roughness (μm)	Breaking Elongation (%)
0	0.31	4.43
10	0.84	10.42
11	0.79	5.04
12	0.81	4.28
13	0.87	7.43
14	0.85	7.30
15	0.82	5.10

**Table 11:** Transmittance of UV topcoat films with different microcapsules added.

Microcapsules (#)	Transmittance (%)
0	87.90
10	83.13
11	77.96
12	79.65
13	78.79
14	79.32
15	82.59

**Table 12:** The glossiness and gloss loss rate of the light source at the incidence angle of 60°.

Microcapsules (#)	Glossiness (GU)	Gloss Loss Rate (%)
0	49.30	-
10	43.40	11.97
11	43.90	10.95
12	43.50	11.76
13	41.90	15.01
14	40.40	18.05
15	37.60	23.73

**Table 13:** Glossiness changes before and after the cold liquid resistance test.

Microcapsules (#)	Glossiness Before Testing (GU)	Glossiness After Testing (GU)	Cold Liquid Resistance Grade (Grade)
0	49.30	43.70	1
10	43.40	34.70	2
11	43.90	38.80	2
12	43.50	26.00	2
13	41.90	26.50	2
14	40.40	23.60	2
15	37.60	22.30	2

**Table 14:** Glossiness and color difference values of the UV topcoat films before and after aging.

Microcapsules (#)	Before Aging Test						After Aging Test					
	Glossiness (GU)	Gloss Loss Rate (%)	*L*	*a*	*b*	Δ*E*	Glossiness (GU)	Gloss Loss Rate (%)	*L*	*a*	*b*	Δ*E*
0	49.30	-	76.93	−2.24	5.08	-	41.00	-	71.20	−0.78	3.69	-
11	43.90	10.95	71.94	−1.95	8.08	5.83	26.30	35.85	69.92	2.15	16.62	13.32

## Data Availability

Data are contained within the article.

## References

[1] Wang C., Yu J.H., Jiang M.H., Li J.Y. (2024). Effect of selective enhancement on the bending performance of fused deposition methods 3D-printed PLA models. BioResources.

[2] Wang C., Zhou Z.Y. (2023). Optical properties and lampshade design applications of PLA 3D printing materials. BioResources.

[3] Zhou J.C., Xu W. (2024). A fast method to prepare highly isotropic and optically adjustable transparent wood-based composites based on interface optimization. Ind. Crops Prod..

[4] Wang C., Zhang C.Y., Ding K.Q., Jiang M.H. (2023). Immersion polishing post-treatment of PLA 3D printed formed parts on its surface and mechanical performance. BioResources.

[5] Wang C., Zhang C.Y., Zhu Y. (2024). Reverse design and additive manufacturing of furniture protective foot covers. Bioresources.

[6] Zhang N., Xu W., Tan Y. (2023). Multi-attribute hierarchical clustering for product family division of customized wooden doors. BioResources.

[7] Gu Y.T., Zhang J.L. (2020). Tensile properties of natural and synthetic rattan strips used as furniture woven materials. Forests.

[8] Hu W.G., Yu R.Z. (2023). Mechanical and acoustic characteristics of four wood species subjected to bending load. Maderas Cienc. Tecnol..

[9] Xue J.X., Xu W., Zhou J.C., Mao W.G., Wu S.S. (2022). Effects of high-temperature heat treatment modification by impregnation on physical and mechanical properties of poplar. Materials.

[10] Wang X.Y., Liu X., Wu S.S., Xu W. (2024). The influence of different impregnation factors on mechanical properties of silica sol-modified Populus tomentosa. Wood Fiber Sci..

[11] Hu W.G., Liu Y., Konukcu A.C. (2023). Study on withdrawal load resistance of screw in wood-based materials: Experimental and numerical. Wood Mater. Sci. Eng..

[12] Hu W.G., Luo M.Y., Hao M.M., Tang B., Wan C. (2023). Study on the effects of selected factors on the diagonal tensile strength of oblique corner furniture joints constructed by wood dowel. Forests.

[13] Liu Y., Hu W.G., Kasal A., Erdil Y.Z. (2023). The state of the art of biomechanics applied in ergonomic furniture design. Appl. Sci..

[14] Hu W.G., Luo M.Y., Yu R.Z., Zhao Y. (2024). Effects of the selected factors on cyclic load performance of T-shaped mortise-and-tenon furniture joints. Wood Mater. Sci. Eng..

[15] Zhou J.C., Xu W. (2025). Optimizing the Interface Compatibility of transparent wood for green phase-change thermal storage. Wood Sci. Technol..

[16] Wu S.S., Zhou L.C., Xu W. (2024). A convenient approach to manufacturing lightweight and high-sound-insulation plywood using furfuryl alcohol/multilayer graphene oxide as a shielding layer. Wood Mater. Sci. Eng..

[17] Hu J., Liu Y., Wang J.X., Xu W. (2024). Study of selective modification effect of constructed structural color layers on European beech wood surfaces. Forests.

[18] Weng M.Y., Zhu Y.T., Mao W.G., Zhou J.C., Xu W. (2023). Nano-Silica/Urea-Formaldehyde Resin-Modified Fast-Growing Lumber Performance Study. Forests.

[19] Liu Q.Q., Gao D., Xu W. (2021). Influence of the Bottom Color Modification and Material Color Modification Process on the Performance of Modified Poplar. Coatings.

[20] Liu Q.Q., Gao D., Xu W. (2022). Effect of Polyurethane Non-Transparent Coating Process on Paint Film Performance Applied on Modified Poplar. Coatings.

[21] Liu C., Xu W. (2022). Effect of Coating Process on Properties of Two-Component Waterborne Polyurethane Coatings for Wood. Coatings.

[22] Zou Y., Zhang M.Y., Li P., Xu B., Zhang Y., Zuo Y.F. (2024). Influence of coating technology on performance of coated wood surface. J. For. Eng..

[23] Zhang W.W., Peng X.X., Li K., Zhang H., Ma J.J., Zhang J.Y. (2024). Aminated modification of shellac resin and properties of its waterborne coatings. J. For. Eng..

[24] Xu R., Wang D., Dou L.H., Cui J.Q., He L., Feng F.Q., Liu F.F. (2024). Preparation and properties of self-healing polyurethane wood coatings with borate eater bonds. J. For. Eng..

[25] Ma L., Xu S.Y. (2024). Investigation on the restoration properties of wood oil microcapsules in wood coatings. Prog. Org. Coat..

[26] Kartsonakis I.A., Kontiza A., Kanellopoulou I.A. (2024). Advanced micro/nanocapsules for self-healing coatings. Appl. Sci..

[27] Chang Y.J., Yan X.X., Wu Z.H. (2023). Application and prospect of self-healing microcapsules in surface coating of wood. Colloid Interfac. Sci..

[28] Zhang H., Cheng C.R., Guo M.L. (2024). Fabrication of diisocyanate microcapsules for self-healing anti-corrosion coatings via integrating electrospraying and interfacial polymerization. Colloid Surf. A Physicochem. Eng. Asp..

[29] Karampoor M.R., Bahrami A., Atapour M. (2024). Towards an antibacterial self-healing coating based on linseed oil/ZnO nanoparticles repair agent, encapsulated in polyurea formaldehyde microcapsules. Micro Nano Lett..

[30] Xia Y.X., Yan X.X. (2024). Preparation of UV topcoat microcapsules and their effect on the properties of UV topcoat paint film. Polymers.

[31] Gu C.Y., Shao J.Q., Liu X.L., Wei J.T., Huang G.Q., Xiao J.X. (2024). Spray drying the Pickering emulsions stabilized by chitosan/ovalbumin polyelectrolyte complexes for the production of oxidation stable tuna oil microcapsules. Int. J. Biol. Macromol..

[32] Lai H.Z., Liu Y., Huang G.T., Chen Y.C., Song Y.G., Ma Y.Q., Yue P.F. (2021). Fabrication and antibacterial evaluation of peppermint oil-loaded composite microcapsules by chitosan-decorated silica nanoparticles stabilized Pickering emulsion templating. Int. J. Biol. Macromol..

[33] Zhang M.C., Li M.Y., Zhang D.Y., Yu Y., Zhu K.X., Zang X.D., Liu D.Y. (2024). Preparation and investigation of sustained-release nanocapsules containing cumin essential oil for their bacteriostatic properties. Food.

[34] Lobato-Guarnido I., Luzón G., Ríos F., Fernández-Serrano M. (2023). Synthesis and characterization of environmentally friendly chitosan-Arabic gum nanoparticles for encapsulation of oregano essential oil in Pickering emulsion. Nanomaterials.

[35] Qian J., Chen Y., Wang Q., Zhao X.H., Yang H.Y., Gong F., Guo H. (2021). Preparation and antimicrobial activity of pectin-chitosan embedding nisin microcapsules. Eur. Polym. J..

[36] Butstraen C., Salaün F. (2014). Preparation of microcapsules by complex coacervation of gum Arabic and chitosan. Carbohydr. Polym..

[37] Baiocco D., Preece J.A., Zhang Z.B. (2021). Microcapsules with a fungal chitosan-gum Arabic-maltodextrin shell to encapsulate health-beneficial peppermint oil. Food Hydrocoll. Health.

[38] Elabbadi A., Erni P. (2020). Gum Arabic/Chitosan Coacervate System.

[39] Wang J.P., Wang J.K., Zhou Q., Li Z., Han Y.S., Song Y., Yang S., Song X.K., Qi T., Möhwald H. (2018). Adaptive polymeric coatings with self-reporting and self-healing dual functions from porous core-shell nanostructures. Macromol. Mater. Eng..

[40] Zhang Z.T., Ye D.Y., Li Y.H., Hu F., Yang Y., Liao Y.W. (2024). Enhancing weatherability and mechanical properties of tung oil wood finishes through natural rubber modification via the Diels-Alder reaction. Int. J. Biol. Macromol..

[41] Samadzadeh M., Boura S.H., Peikari M., Ashrafi A., Kasiriha M. (2011). Tung oil: An autonomous repairing agent for self-healing epoxy coatings. Prog. Org. Coat..

[42] Chang Y.J., Wu Z.H., Liu E.W. (2024). Fabrication of chitosan-encapsulated microcapsules containing wood wax oil for antibacterial self-healing wood coatings. Ind. Crop. Prod..

[43] (2013). Physical and Chemical Performance Tests of the Paint Film on the Surface of Furniture—Part 6: Method for Determining Gloss.

[44] (2021). Methods for Measuring the Colour of Paint Films—Part I: Cold Liquid Resistance Measurement Method.

[45] (2008). Paints and Varnishes—Rating Schemes of Degradation of Coats.

[46] Beirao-da-Costa S., Duarte C., Bourbon A.I., Pinheiro A.C., Januário M.I.N., Vicente A.A., Beirao-da-Costa M.L., Delgadillo I. (2013). Inulin potential for encapsulation and controlled delivery of Oregano essential oil. Food Hydrocoll..

[47] Yan X.X., Tao Y., Chang Y.J. (2021). Effect of Shellac Waterborne Coating Microcapsules on the Optical, Mechanical and Self-Healing Properties of Waterborne Primer on Tilia European L.. Wood. Coat..

[48] Peng W.W., Yan X.X. (2022). Preparation of Tung Oil Microcapsule and Its Effect on Wood Surface Coating. Polymers.

[49] Zhang H., Zhou L.M., Shehzad H., Farooqi Z.H., Sharif A., Ahmed E., Habiba U., Qaisar F., Noor E.-F., Begum R. (2024). Innovative free radical induced synthesis of WO_3_^–^ doped diethyl malonate grafted chitosan encapsulated with phosphorylated alginate matrix for UO_2_^2+^ adsorption: Parameters optimisation through response surface methodology. Sep. Purif. Technol..

[50] Baiocco D., Preece J.A., Zhang Z.B. (2021). Encapsulation of hexylsalicylate in an animal-free chitosan-gum Arabic shell by complex coacervation. Colloid Surf. A.

[51] Shukla A., Syaifie P.H., Rochman N.T., Syaifullah S.J., Jauhar M.M., Mardliyati E. (2025). A recent study of natural hydrogels: Improving mechanical properties for biomedical applications. Biomed. Mater..

